# Interaction between functionalized graphene and sulfur compounds in a lithium–sulfur battery – a density functional theory investigation†

**DOI:** 10.1039/c7ra11628d

**Published:** 2018-01-09

**Authors:** Kimal Chandula Wasalathilake, Md Roknuzzaman, Kostya (Ken) Ostrikov, Godwin A. Ayoko, Cheng Yan

**Affiliations:** School of Chemistry, Physics and Mechanical Engineering, Faculty of Science and Engineering, Queensland University of Technology (QUT) Brisbane QLD 4001 Australia c2.yan@qut.edu.au

## Abstract

Lithium–sulfur (Li–S) batteries are emerging as one of the promising candidates for next generation rechargeable batteries. However, dissolution of lithium polysulfides in the liquid electrolyte, low electrical conductivity of sulfur and large volume change during electrochemical cycling are the main technical challenges for practical applications. In this study, a systematic first-principles density functional theory calculation is adopted to understand the interactions between graphene and graphene with oxygen containing functional groups (hydroxyl, epoxy and carboxyl groups) and sulphur (S_8_) and long chain lithium polysulfides (Li_2_S_8_ and Li_2_S_4_). We find the adsorption is dominated by different mechanisms in sulphur and lithium polysulfides, *i.e.* van der Waals attraction and formation of coordinate covalent Li–O bonds. The adsorption strength is dependent on the inter-layer distance and electron rich functional groups. Through these mechanisms, sulphur and lithium polysulfides can be successfully retained in porous graphene, leading to improved conductivity and charge transfer in the cathode of Li–S batteries.

## Abstract 2

Functionalized graphene can successfully anchor sulfur compounds *via* moderate interactions, leading to improved conductivity and charge transfer in the cathode of Li–S batteries.

## Graphical abstract

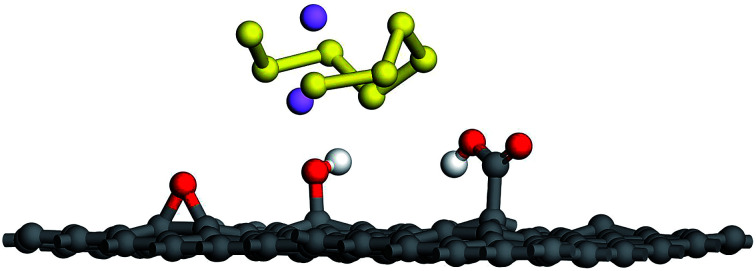

## Introduction

Recently, there has been an increasing demand for higher energy density in energy storage systems such as batteries and supercapacitors for portable devices and electric vehicles. With an energy capacity of 1673 mA h g^−1^ and a specific energy of 2600 W h kg^−1^, Li–S batteries are emerging as one of the promising candidates for next generation rechargeable batteries.^1–3^ However, dissolution of lithium polysulfides in the liquid electrolyte, low electrical conductivity of sulfur and its final discharge products, and large volume change during electrochemical cycling are the main technical challenges.^4,5^ To solve these problems, recent efforts have been put into the design of nanostructured electrodes to improve their capacity and cycling performance.^6,7^ It has been confirmed that composite cathodes consisting of sulfur and nanostructured carbon materials such as meso-micro porous carbon,^8^ carbon spheres^9,10^ carbon nanotubes (CNTs),^11^ graphene,^12–15^ and graphene oxide,^16,17^ could mitigate the polysulfide shuttle *via* physical confinement of soluble polysulfides within the conductive carbonaceous structures. Among the various types of carbon materials, graphene has attracted much attention due to its good electrical conductivity and high surface area. Another alternative is reduced graphene oxide (rGO), a layered material with graphene domains and residual functional groups such as hydroxyl, epoxy and carboxyl groups.^18,19^ Consequently, attempts were made to design cathodes with porous graphene and rGO.^20–23^ Chen's group^24^ employed a three-dimensional rGO sponge to produce a sulfur nanogranular film-coated composite cathode with a reversible capacity of 1080 mA h g^−1^ at 0.1C rate and 86.2% capacity retention after 500 cycles at 1.0C rate.

In General, microporous anchoring materials (pore size < 2 nm) could successfully confine polysulfides to achieve good electro activity and long-term cyclability.^25–27^ However it was realized that anchoring materials with non-polar surfaces, alone cannot mitigate the polysulfide shuttle as they fail to make sufficient interaction with polar lithium polysulfides, and further surface modification is generally needed to chemically bind polysulfides onto the carbonaceous matrix. Recently, density functional theory (DFT) studies were carried out to investigate the discharge mechanisms in Li–S batteries,^28,29^ and several investigators modelled the interactions between defective graphene, heteroatom doped graphene, and lithium polysulfides.^30–32^ However, the anchoring mechanisms of porous graphene functionalized with different oxygen groups during lithiation process have not been well understood. In addition, it is unclear if polar groups can facilitate the adsorption of non-polar S_8_ as they interact with lithium polysulfides and if the functional groups can build up a barrier for electron transfer at the interface between S_8_ and the substrate.

In this paper, we report a systematic first-principle investigation on the anchoring effects of microporous epoxy-, hydroxyl- and carboxyl-functionalized graphene and elucidate the mechanisms responsive for interfacial interaction and electron transfer.

### Computational methods

**Fig. 1:**
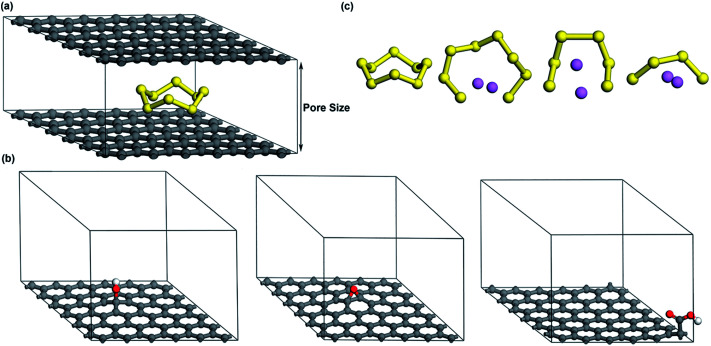
(a) Microporous graphene (b) microporous structures of hG, eG and cG respectively (c) geometrically optimized minimum energy structures of S_8_, Li_2_S_8_, Li_2_S_6_ and Li_2_S_4_ respectively (carbon, sulfur, oxygen, hydrogen and lithium atoms are represented by grey, yellow, red, white and purple, respectively).

As shown in Fig. 1a, a basic structure with two parallel graphene layers is constructed to simulate porous graphene. The interlayer distance is changed in the range of 7.5–20 Å to understand how the pore size affects the adsorption of S compounds. The smallest interlayer distance (pore size) is set to 7.5 Å to effectively accommodate the dimensions of cyclo-S_8_ (∼0.7 nm).^33,34^ To model oxygen functionalized graphene (OFG) which consists of hydroxyl, epoxy and carboxyl functional groups, three distinct microporous structures are built using the basic model shown in Fig. 1a with a interlayer distance of 12.5 Å. For simplicity, they are referred as hG, eG and cG, respectively (Fig. 1b). Based on literature,^35,36^ hydroxyl and epoxy groups are introduced on the basal plane and a carboxyl group is introduced at the edge of the plane corresponding to oxygen atomic concentration of 1.4–2.6%.

Molecule configurations of S compounds, adsorption energies of S compounds to microporous structures (graphene and OFG), charge transfer from S compounds at different lithiation stages, and density of states near Fermi energy region are examined using DFT calculations with DMol3 package^37,38^ of Materials Studio 2016. Electron–electron exchange correlations are described by generalized gradient approximations (GGA) of the Perdew–Burke–Ernzerhof (PBE) functional.^39^ To consider the van der Waals interaction, a semi-empirical dispersion potential of DFT-D2 method of Grimme is used.^40^ Energy, maximum force and maximum displacement are set to 2.0 × 10^−5^ ha, 4 × 10^−3^ ha A^−1^ and 5 × 10^−3^ A, respectively. Self-consistent-field density is set to 1 × 10^−5^ eV. Double numerical plus polarization (DNP) is selected as the basis set and effective core potentials (ECP) are employed to describe the core electrons. The corresponding *k* point grid is generated by the Monkhrost–Pack technique^41^ for the Brillouin zone sampling and simulations are carried out using a 5 × 5 × 1 grid.

The binding energy (*E*_bind_) of each Li_2_S_*x*_ molecules can be calculated as, *E*_bind_ = [*E*_Li_2_S_*x*__ − (2*E*_Li_ + *E*_S_*x*__)]/2, where *E*_Li_2_S_*x*__, *E*_Li_ and *E*_S_*x*__ are the energies of Li_2_S_*x*_, Li atom and S_*x*_ molecule respectively. The adsorption energy (*E*_ads_) of each S species to various graphene structures are calculated according to, *E*_ads_ = *E*_Total_ − (*E*_graphene_ + *E*_S_), where *E*_Total_, *E*_graphene_ and *E*_S_ are the total energies of the system, graphene structure and S containing cluster (S_8_, Li_2_S_8_ or Li_2_S_4_), respectively. According to the equation, more negative adsorption energy indicates stronger interaction between graphene structure and S cluster. Initially, sulfur species were placed with different orientations inside the graphene structure. Eight different potential configurations of each S compound were considered and after relaxation, corresponding adsorption values were compared to obtain the most stable configuration (Fig. S1†). The energy difference between intact structure (Li_2_S_8_/Li_2_S_4_) and decomposed structure (Li + LiS_8_/Li + LiS_4_) is calculated according to, Δ*E* = *E*(Li_2_S_*x*_ + graphene) − *E*(Li + LiS_*x*_ + graphene), where *x* = 4 and 8.

## Results and discussion

### Effect of microporous graphene on adsorption of S species

To find the structures with the minimum energy in S_8_, Li_2_S_8_, Li_2_S_6_ and Li_2_S_4_, geometry optimization was carried out to obtain possible linear or closed atomic arrangements.^30,42,43^Fig. 1c shows the stable ground state structures of S_8_ and lithium polysulfides. Their bond lengths are consistent with previous investigations.^30^ The calculated bond length, charge of each atom *via* the Mulliken population analysis (MPA) and the binding energies of lithium polysulfides are shown in Table 1.

**Table 1:** Average bond distances, average charges and binding energies

	Li_2_S_8_	Li_2_S_6_	Li_2_S_4_
Li–S (Å)	2.450	2.429	2.405
S–S (Å)	2.103	2.138	2.116
Charge on Li	0.44	0.44	0.46
Binding energy (eV)	−3.39	−4.03	−3.50

The most stable configuration of sulfur is the cycloocta-S which consists of covalently bonded 8 sulfur atoms in a crown formation with a calculated average S–S bond length of 2.092 Å. During the initial phase of the discharge process, S_8_ is reduced to S_8_^2−^, S_6_^2−^, S_4_^2−^ forming high-order lithium polysulfides and further gets reduced to S_2_^2−^ and S^2−^ forming low-order lithium polysulfides at the latter stages.^2^ According to our calculations, as *x* in Li_2_S_*x*_ decreases from 8 to 4, the average Li–S distance decreases from 2.450 Å to 2.405 Å, implying that the interaction between Li and S atom strengthens as the *x* decreases. The average S–S bond lengths of Li_2_S_8_, Li_2_S_6_ and Li_2_S_4_ are 2.103, 2.138 and 2.118 Å, respectively. Our results revealed that S–S bond lengths of polysulfides are larger than that of S_8_ molecule which is 2.092 Å due to the less covalent nature of the S cluster within the lithium polysulfide. Due to the weak interaction between Li and S, high order polysulfides tend to ionize easily into Li and polysulfide ions in the electrolyte than low-order Li_2_S_2_ and Li_2_S. MPA is a qualitative method to understand how the charge is distributed among each atom. According to MPA, the average net charge of Li is 0.44, 0.44 and 0.46 in Li_2_S_8_, Li_2_S_6_ and Li_2_S_4_ respectively. Therefore, the interaction between Li and S in Li_2_S_8_ is the weakest due to the largest Li–S distance and the lowest average net charge of Li. Moreover, Li_2_S_8_ exhibits the lowest binding energy of −3.39 eV, suggesting that it is the most soluble compound among these lithium polysulfides during a charge/discharge cycle, consistent with previous experiments.^44^ As an intermediate which presents in both high and low voltage plateau regimes of charge/discharge cycle, Li_2_S_4_ plays a vital role in the Li–S redox reaction. Elemental S is the starting point of the multi-electron-transfer cathode reaction, and hence the retention of S_8_ is important for the long lasting performance of a Li–S cell. For these reasons, the interaction between S_8_, Li_2_S_8_ and Li_2_S_4_ and the porous graphene deserves further investigation.

**Fig. 2:**
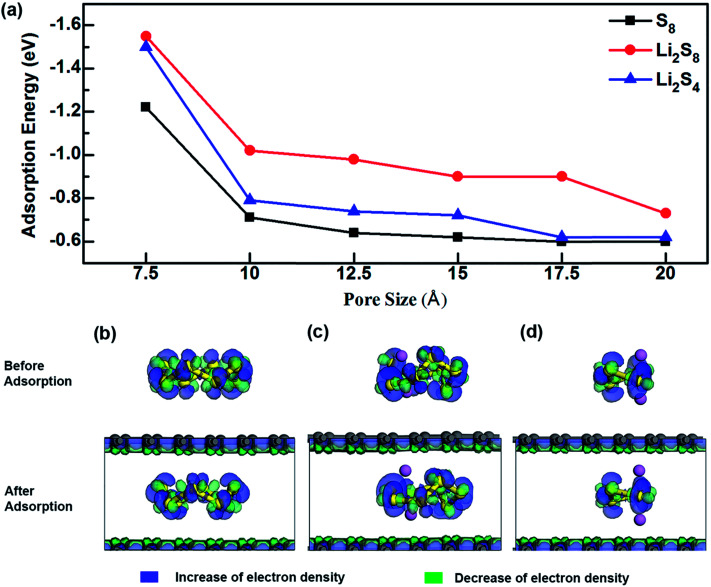
(a) Adsorption energies of S_8_, Li_2_S_8_ and Li_2_S_4_ to microporous graphene. Deformation charge density and adsorption site of (b) S_8_, (c) Li_2_S_8_, and (d) Li_2_S_4_.

As shown in Fig. 2a (Table S1†), the variation of energy profiles indicates that the adsorption energy (*E*_ads_) increases as the pore size decreases. The adsorption energy of S compounds to a typical anchoring material of the cathode depends on (1) the chemical interaction between the lithium polysulfide and the anchoring material in which a covalent bond can be formed between the Li atom in Li_2_S_*x*_ and the functional group of the anchoring material and/or between the S atom of Li_2_S_*x*_ and the functional group of the anchoring material and (2) the physical van der Waals attraction. Unlithiated S_8_ is a non-polar molecule, and does not form any chemical interaction with graphene domains. Consequently, the interfacial interaction is mainly governed by the physical interaction. However, as the lithiation begins, apart from the physical interaction, a chemical interaction between Li atoms and the anchoring material is formed. In graphene, regardless of different lithiation stages the physical interaction overpowers the chemical interaction and there is a significant increase of adsorption strength as the pore size decreases from 10 Å to 7.5 Å. When the pore size is 7.5 Å, the adsorption energies are −1.55 eV, −1.50 eV and −1.22 eV for Li_2_S_8_, Li_2_S_4_ and S_8_, respectively.

The effect of van der Waals interaction on the confinement of S species inside microporous graphene can be visualized from the deformation charge density analysis (Fig. 2b–d), in which the increase and decrease of total electron density are denoted in blue and green respectively. The electron density difference is expressed as, Δ*ρ* = *ρ*_Total_ − (*ρ*_graphene_ + *ρ*_S_), where *ρ*_Total_, *ρ*_graphene_ and *ρ*_S_ are the electron densities of the system, graphene structure and the S-containing cluster respectively. It can be seen that charge is transferred inside the S species and inside the graphene surfaces, but no apparent charge transfer occurs between them, suggesting no strong chemical interaction. However, by thoroughly comparing the deformation charge density of Li_2_S_8_ and Li_2_S_4_ with their adsorption sites (Fig. 2c–d), it can be seen that Li atoms in S complexes have slightly moved away from S and towards the graphene surface, indicating a slight chemical attraction between Li and C atoms which explains why Li_2_S_8_ and Li_2_S_4_ exhibit higher adsorption energy values than S_8_. Due to the fact that unlithiated S_8_ is confined inside a narrow pore which induces a strong physical interaction from both sides of the graphene surfaces, the adsorption value of −1.22 eV exhibited by microporous graphene with a pore size of 7.5 Å is much higher than values recorded for anchoring materials like V_2_O_5_, MoS_2_ and phosphorene.^45,46^

### Influence of different functional groups of OFG towards adsorption of S species

**Fig. 3:**
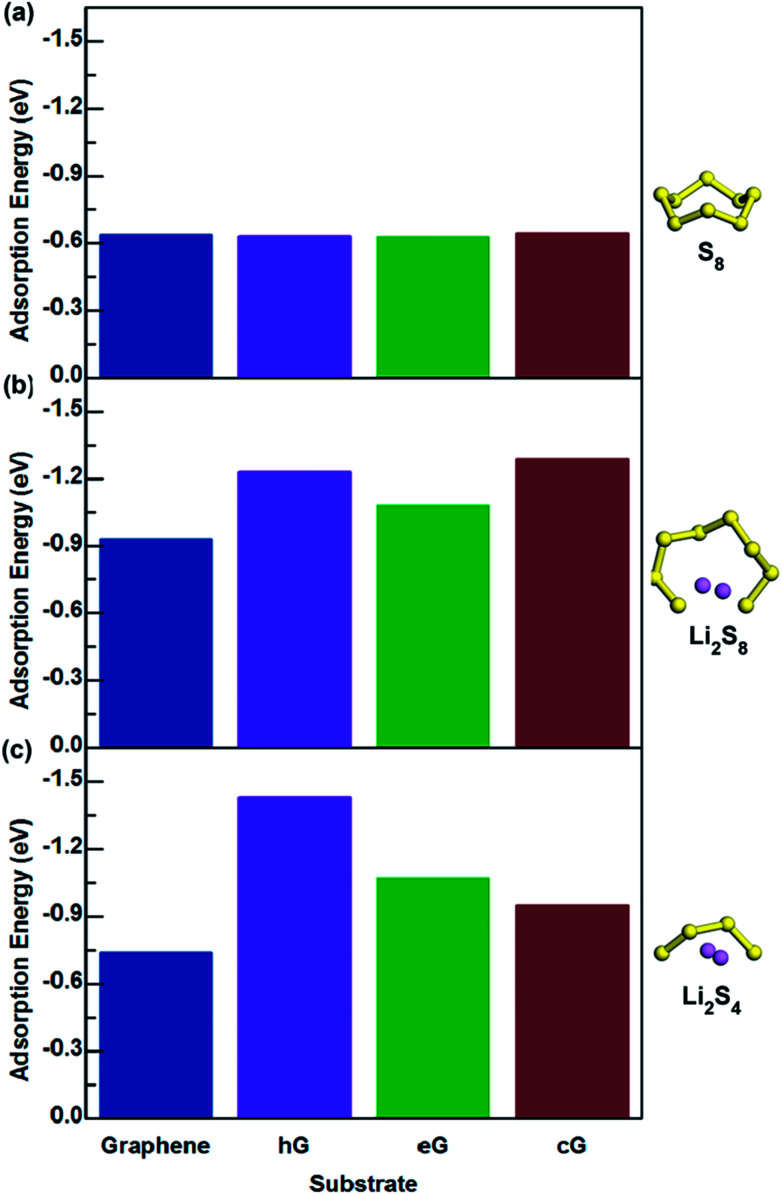
Adsorption values of (a) S_8_, (b) Li_2_S_8_, and (c) Li_2_S_4_ interacting with graphene, hG, eG and cG.

**Fig. 4:**
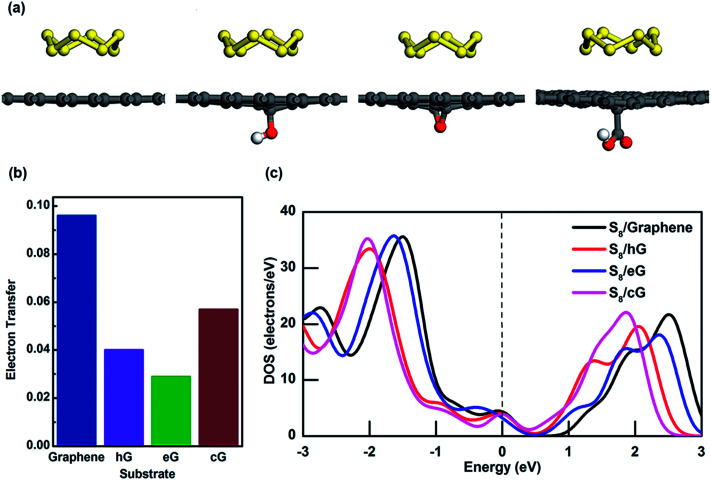
(a) S_8_ adsorption on graphene, hG, eG and cG. Oxygen and hydrogen atoms are represented by red and white respectively. (b) Electron transfer from S_8_ to different substrates, and (c) density of states near Fermi energy region for S_8_ on graphene, hG, eG and cG.

The interactions between S_8_, Li_2_S_4_ and Li_2_S_8_ molecules with functional groups hG, eG and cG, were simulated and the adsorption energies are summarized in Fig. 3 (Table S2†). Fig. 4a shows the final optimized structures of S_8_ on pure graphene, hG, eG and cG, respectively. Note that in all the cases, the highest adsorption energy was given when S_8_ adsorbed from the opposite direction of the functional group and parallel to the graphene surface. According to our calculations, it was found that when compared with graphene, hG, eG and cG exhibit almost similar adsorption energy to S_8_, illustrating that oxygen functional groups do not have a major influence on the adsorption of S_8_, as the adsorption strength is dominated by van der Waals attraction.

To further investigate the interaction between S_8_ and porous graphene, MPA is applied to determine how much charge transfer has occurred between them. Interestingly, 0.09 electrons have been transferred from S_8_ to the graphene surface but there is only a minor contribution to charge transfer between S_8_ and OFG where only 0.04, 0.03 and 0.06 electrons have been withdrawn from hG, eG and cG respectively (Fig. 4b). Fig. 4c shows the density of states (DOS) near Fermi energy (*E*_f_) for the adsorption system of S_8_ in graphene and OFG, in which the *E*_f_ is set to 0 eV and represented by a vertical dotted line. For S_8_ adsorbed graphene system, the DOS value at *E*_f_ is found to be 4.33 electrons per eV and it reduces to 4.00 and 3.92 electrons per eV when S_8_ gets adsorbed to hG and cG, respectively (Table S3†). Furthermore DOS value at *E*_f_ drops to 3.15 electrons per eV when eG adsorbs S_8_, suggesting that electron transfer at the interface is slightly hindered due to the oxygen functional groups on the graphene surface which then leads to building up of ohmic resistance at the S_8_/OFG interface. This phenomenon is also reported by Shiqi *et al.* where they discovered that S_8_ is decoupled from the graphitic surface due to the presence of Triton X-100, a non-ionic surfactant with a polyethylene oxide chain.^47^

**Fig. 5:**
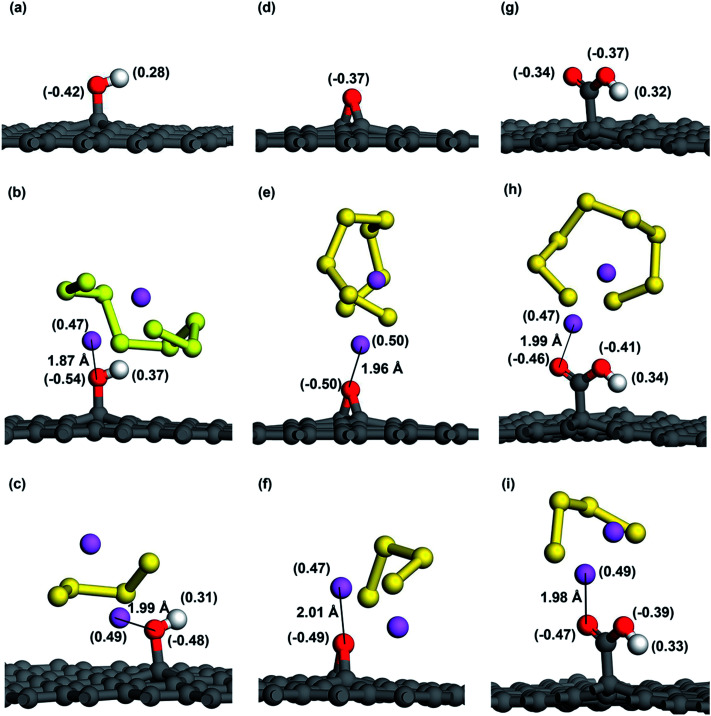
Mulliken charge distribution before and after adsorption of Li_2_S_8_ and Li_2_S_4_ of (a–c) hG, (d–f) eG and (g–i) cG respectively. Note that charge of each atom is shown in parentheses. The shortest distance between Li atom and O atom is also shown in angstroms (Å).

As for the adsorption of Li_2_S_8_ molecule, cG exhibits the highest adsorption energy of −1.29 eV and all the oxygen functional groups show significantly higher adsorption energies compared to pristine graphene (Fig. 3b). When interacting with Li_2_S_4_, hG exhibits the highest adsorption energy of −1.43 eV and it is almost over 2 times higher than pristine graphene (Fig. 3c). Fig. 5b–i shows the optimized structures of Li_2_S_8_ and Li_2_S_4_ on hG, eG and cG, respectively. To further get an understanding of the magnitude of the Li–O interaction, we optimized the geometry of a Li_2_O crystal (Fig. S2†) so that it could be used as a benchmark to compare the bonding nature of Li and O in the optimized models.^48^ The calculated Li–O distances of adsorption geometries are almost similar with the strong ionic bond length of Li_2_O molecule implying the existence of a strong attraction between Li and O in all the cases. hG had the shortest Li–O distance of 1.87 Å when it interacted with Li_2_S_8_ while the longest Li–O distance of 2.01 Å was given by eG when it interacted with Li_2_S_4_ (Table S4†).

**Fig. 6:**
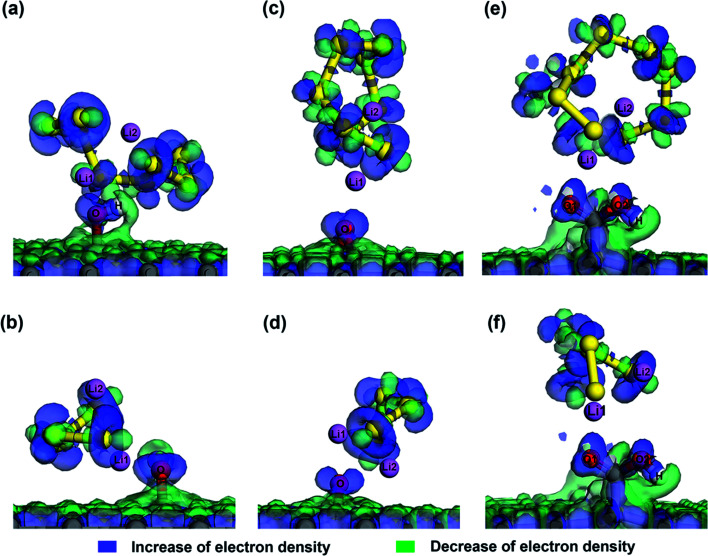
Deformation charge density at Li_2_S_8_ and Li_2_S_4_ adsorption sites of (a and b) hG, (c and d) eG and (e and f) cG. (The increase/decrease of electron density is denoted by blue/green, respectively.)

As O atom has a high electronegativity of 3.44 and Li atom has a low electronegativity of 0.98, the strong attraction between Li and O can be explained by the Lewis-acid base theory. Since epoxy, hydroxyl and carboxyl groups all consist of O atoms with lone electron pairs in their outer p orbitals; they act as electron pair donors (Lewis bases). These Lewis-base sites are attracted by the terminal Li atoms of Li_2_S_8_ and Li_2_S_4_ which act as strong Lewis acids according to the Lewis-acid base theory. Fig. 6 shows the deformation charge density corresponding to Li_2_S_8_ and Li_2_S_4_ adsorption sites. A significantly high electron density is visible around the lone pairs of the O atom strengthening the fact that extra pairs of electrons act as electron rich donor to interact with strong Lewis acid of Li ion to form a coordinate covalent bond. The strong attraction between negatively charged oxygen atom and the positively charged Li atom can be further illustrated in Fig. 5 where the Mulliken charge distribution of hG, eG and cG before and after adsorption of Li_2_S_8_ and Li_2_S_4_ are presented. Because of the polarization of the O atom by the terminal Li atom of Li_2_S_8_, 0.12, 0.13 and 0.12 electrons are withdrawn by the closest O atom of each eG, hG and cG respectively. The same phenomenon occurs when those substrates interact with Li_2_S_4_ where 0.06, 0.12 and 0.13 electrons have been transferred to the O atom. Therefore functionalized carbon materials consisting of highly electronegative atoms with lone electron pairs, are good candidates for the immobilization of high order lithium polysulfides due to the moderate adsorption ability. Recently, a composite cathode made out of chlorine-reinforced carbon nanofibers reported to have enhanced cycling performance, validating our theoretical predictions.^49^

Though there is a relatively strong interaction between lithium polysulfides and the OFG, the polysulfide molecule itself remains intact without being dissociated into short chain lithium polysulfides upon adsorption. We observed that after adsorption, the internal Li–S bond length increased slightly from 2.45 Å to 2.48 Å in Li_2_S_8_ and 2.41 Å to 2.46 Å in Li_2_S_4_ (Table S4†). Various layered metal oxides and sulfides were reported to induce a strong chemical bond with lithium polysulfides in terms of Li–O, Li–S or M–S bonds (M represents metal oxides/sulfides).^50,51^ However, recently it was discovered that strong interaction with polysulfides could interfere on S reduction reactions and sometimes weaken the Li–S bond causing the dissociation of the Li_2_S_*x*_ molecule. Such separation between Li and S atom eventually could lead up to the formation of Li^+^ and S_*x*_^2−^ ions and as a result sulfur could be dissolved in the electrolyte adversely affecting the performance of the Li–S cell.^49,50,52^ Furthermore, Yu *et al.* revealed the importance of employing a cathode material with a moderate binding capability which allows a small amount of polysulfide dissolution in order to improve the stability of the solid electrolyte interface (SEI) on the lithium anode.^49^

**Fig. 7:**
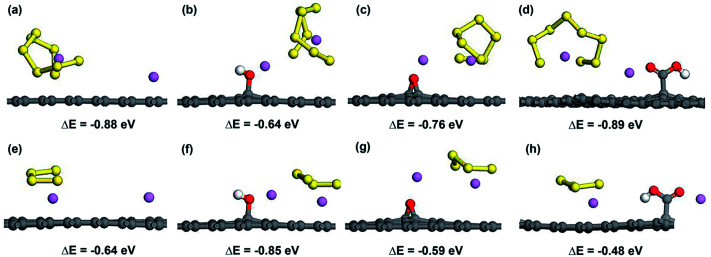
Atomic configurations and Δ*E* values of Li + LiS_8_ and Li + LiS_4_ clusters adsorbed on graphene (a and e), hG (b and f), eG (c and g) and cG (d and h) respectively.

To further investigate the possibility of the decomposition of Li_2_S_*x*_ by the weakening of the Li–S bond due to the attraction of OFG, we considered the adsorption of the decomposed LiS_8_ and LiS_4_ structures along with an isolated Li atom and calculated the energy difference (Δ*E*) between the decomposed structure and the intact structure (Li_2_S_8_ and Li_2_S_4_). A negative value for Δ*E* indicates that the intact structure has lower energy than the decomposed structure and the intact structure is energetically stable. Fig. 7 shows the adsorbed Li + LiS_8_ and Li + LiS_4_ clusters on graphene, hG, eG and cG. According to Δ*E* values in all the cases, the intact structure proved to be energetically preferable over the decomposed structure. Therefore microporous graphene decorated with oxygen functional groups could be considered as an effective choice as a cathode material for Li–S batteries which strikes a balance between adsorption strength and intactness of high order lithium polysulfides.

To gain further insights into the bond interaction between OFG and Li_2_S_8_/Li_2_S_4_, we analysed the atomic partial density of states (PDOS) near *E*_f_ for the adsorption systems (Fig. S3†). The PDOS for Li-1 and O-1 atoms (indicated by black and blue colour) are seen to overlap at the upper part of the valence band (just below the Fermi level) suggesting that there is a hybridization between Li-2s and O-2p orbitals. This further ascertains the existence of a Li–O covalent bond which enhances the interaction between the substrate and the polysulfides and thereby mitigates the polysulfide shuttle effectively.

The DOS near Fermi energy for hG, eG and cG before and after the anchoring of S_8_, Li_2_S_8_ and Li_2_S_4_ are presented in Fig. S4,† in which hG, eG and cG exhibit metallic nature with 3.98, 3.16 and 3.92 electrons per eV DOS values at *E*_f_ respectively at oxygen atomic concentrations of 1.4–2.6%. The good electrical conductivity of reduced graphene oxide has also been demonstrated by previous computational studies^53,54^ and, furthermore Stankovich *et al.*^55^ produced rGO nanosheets with a significantly high conductivity value of (∼2 × 10^2^ S m^−1^) which closely approaches that of pristine graphene, even at an atomic C/O ratio of ∼10.

The strong affinity towards Li_2_S_8_/Li_2_S_4_ by the OFG is clearly observed from the DOS values of hG, eG and cG after anchoring of Li_2_S_8_ and Li_2_S_4_. When Li_2_S_8_ and Li_2_S_4_ are adsorbed, due to the newly formed Li–O covalent bond between Li atom of Li_2_S_8_/Li_2_S_4_ and O atom of the functional groups, more electrons are transferred to the adsorption sites and the DOS curve exhibits a higher value at *E*_f_ when compared with the S_8_ adsorbed system. Therefore polar groups maintain a strong interaction with the lithium polysulfides in the discharge/charge cycle of the Li–S battery and in the meantime improve the electrical conductivity of the cathode by facilitating the charge transfer at the interface.

**Fig. 8:**
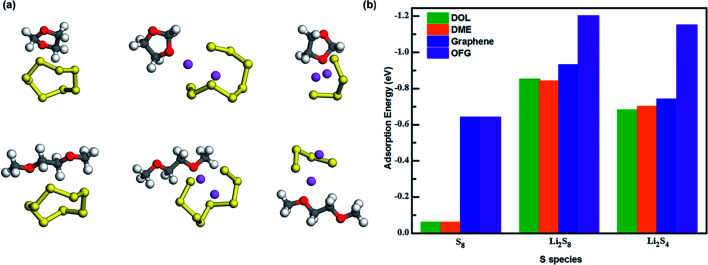
(a) Interaction of S_8_, Li_2_S_8_ and Li_2_S_4_ with DOL and DME. (b) Adsorption energies of S_8_, Li_2_S_8_ and Li_2_S_4_ interacting with DOL, DME, graphene and OFG.

In a typical Li–S battery, the organic electrolyte consists of bis(trifluoromethane)sulfonimide lithium salt (LiTFSI) dissolved in a mixture of 1,3-dioxolane (DOL) and 1,2-dimethoxyethane (DME). To investigate the influence of the electrolyte towards the discharge/charge cycle of the battery, we calculated the adsorption energy values of DOL and DME towards S_8_, Li_2_S_8_ and Li_2_S_4_ (Fig. 8a) and compared them with the values we gained for graphene and OFG. For ease of comparison, we used the average adsorption values of hG, eG, and cG as the adsorption value for OFG in each lithiation stage. According to Fig. 8b, adsorption energies of graphene and OFG towards S_8_ are almost similar and they are significantly higher than DOL and DME, suggesting that van der Waals interaction is more than sufficient to retain S_8_ in the cathode. However Li_2_S_8_ and Li_2_S_4_ are barely adsorbed by graphene and there's a high possibility of these long chain polysulfides being dissolved in the liquid electrolyte. When compared with DOL and DME, adsorption energy of OFG towards Li_2_S_8_/Li_2_S_4_ is significantly high proving the fact that functional groups could confine lithium polysulfides inside the cathode and mitigate the shuttle effect.

Based on the insights gained from our theoretical study, we can conclude that adsorption strength and electron transfer at the interface are two critical aspects which have to be considered when selecting an anchoring material for the cathode in Li–S batteries. At the initial stage of the discharge cycle, confinement of S_8_ is controlled by the van der Waals interaction, and it is highly unlikely for S_8_ being dissolved in the electrolyte due to the poor attraction of the solvents towards S_8_ molecules. A microporous substrate is more suitable to confine S_8_ as it can be confined inside a narrow pore which induces a strong physical interaction. Surface functionalization of graphene has no additional influence on attracting S_8_ to its surface since almost no chemical interaction can be formed between functional groups and non-polar S_8_. Furthermore functional groups adversely affect the electron transfer and increase the ohmic resistance at the substrate/S_8_ interface. However functional groups have a major impact on mitigating the shuttle effect and improving the capacity retention of Li–S cell by ensuring a strong interaction with Li_2_S_8_/Li_2_S_4_. On the other hand, the strong interaction with lithium polysulfides facilitates the interfacial electron transfer through the Li–O bond which can be explained by the Lewis-acid base theory.

## Conclusions

In this work, we have investigated the interactions between graphene with oxygen containing functional groups (hydroxyl, epoxy and carboxyl groups) and sulphur (S_8_) and long chain lithium polysulfides (Li_2_S_8_ and Li_2_S_4_), *via* density functional theory computations. During the initial unlithiated stage, we find the interaction between sulphur and pristine graphene and OFG are dominated by van der Waals attraction. Although graphene and OFG exhibits almost the same adsorption capability towards non-polar S_8_, the functional groups develop a slight barrier for electron transfer at the interface and increase the ohmic resistance. The highest adsorption energy is observed when the distance between two graphene layers approaches to about 7.5 Å. During the lithiation stage, surface functionalization of graphene significantly enhances the interaction with Li_2_S_8_/Li_2_S_4_ by forming a coordinate covalent Li–O bond. Due to the covalent nature of the Li–O bond, polysulfides are well retained inside the cathode and it also improves the conductivity of the electrode upon the deposition of Li_2_S_8_ and Li_2_S_4_ by facilitating the interfacial charge transfer. Furthermore, our work explains the reason why porous graphene with oxygen functional groups is more effective as a cathode material compared to materials with too strong interactions which could cause destruction effects on the adsorbed lithium polysulfides. Due to the moderate binding affinity, OFG strikes a balance between adsorption strength and intactness of high order lithium polysulfides. Therefore based on our simulations, we suggest that microporous graphene decorated with hydroxyl, epoxy and carboxyl functional groups can successfully anchor sulfur and lithium polysulfides *via* moderate interactions, leading to improved conductivity and charge transfer in the cathode of Li–S batteries.

## Conflicts of interest

The authors declare no conflict of interest.

## Supplementary Material

RA-008-C7RA11628D-s001
