# Supplementary material for: Interaction between functionalized graphene and sulfur compounds in a lithium–sulfur battery – a density functional theory investigation
Source: RSC Adv. 2018 Jan 9;8(5):2271–9. doi: 10.1039/c7ra11628d (PMC9077337; doi:10.1039/c7ra11628d)
Supplement: RA-008-C7RA11628D-s001 [file RA-008-C7RA11628D-s001.pdf]

Electronic Supplementary Material (ESI) for RSC Advances.
This journal is © The Royal Society of Chemistry 2017

Supporting Information for

Interaction between functionalized graphene and sulfur compounds in lithium-sulfur battery – a density functional theory investigation

Kimal Chandula Wasalathilake[a], Md Roknuzzaman[a], Kostya (Ken) Ostrikov[a], Godwin A. Ayoko[a] and Cheng Yan*[a]

[a]School of Chemistry, Physics and Mechanical Engineering, Faculty of Science and Engineering, Queensland University of Technology (QUT), Brisbane, QLD 4001, Australia

*Email: c2.yan@qut.edu.au

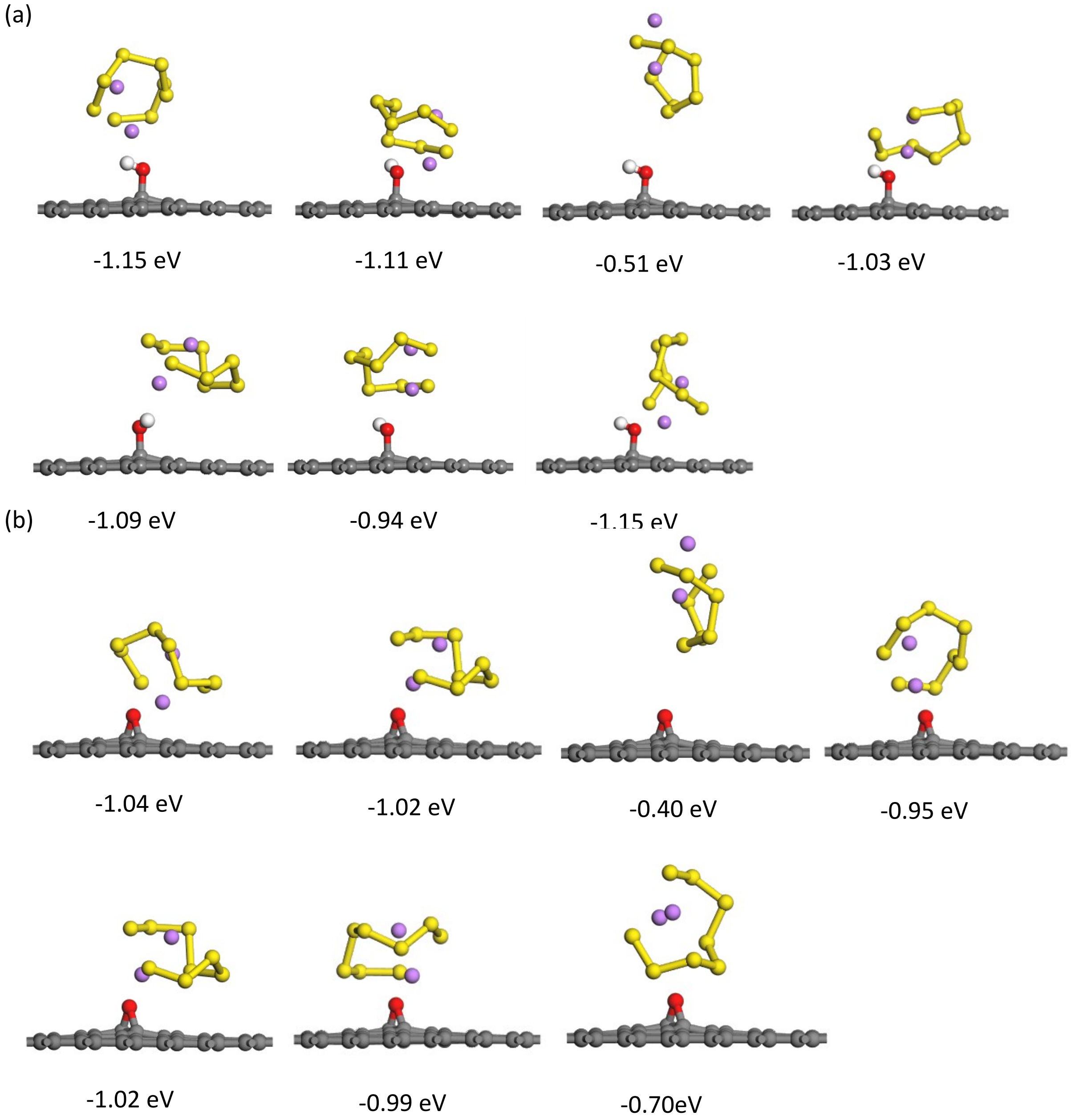

(c)

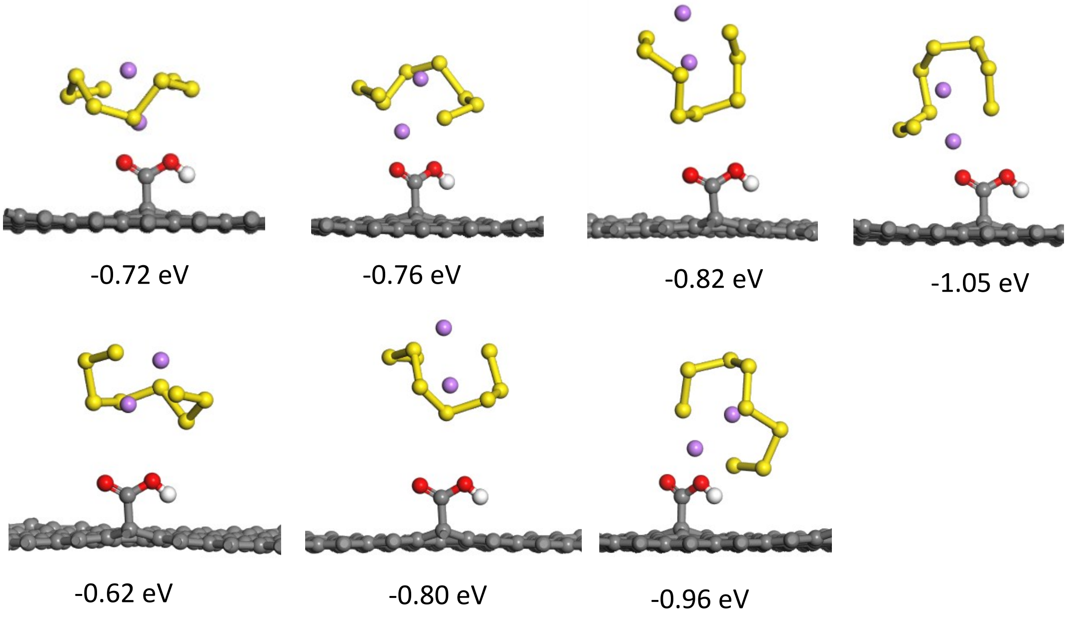
-0.72 eV
-0.76 eV
-0.82 eV
-1.05 eV
-0.62 eV
-0.80 eV
-0.96 eV

(d)

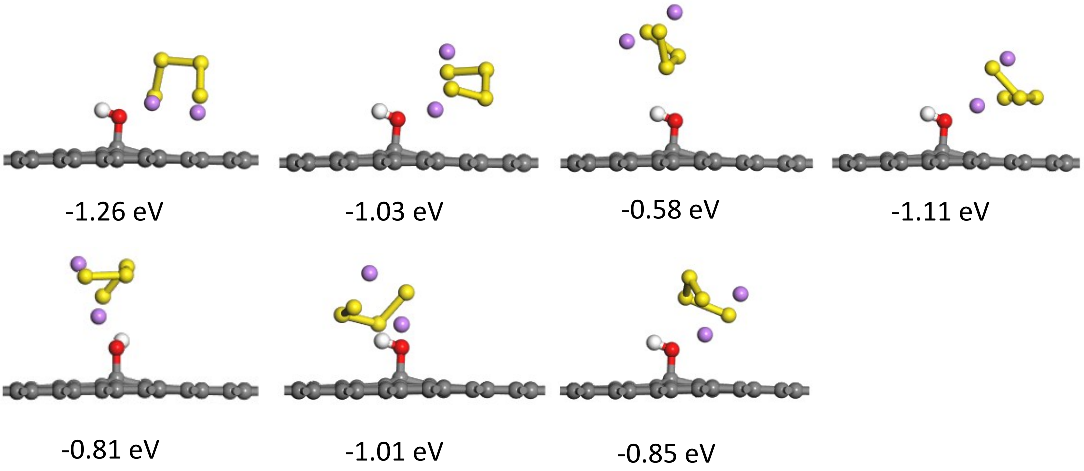
-1.26 eV
-1.03 eV
-0.58 eV
-1.11 eV
-0.81 eV
-1.01 eV
-0.85 eV

(e)

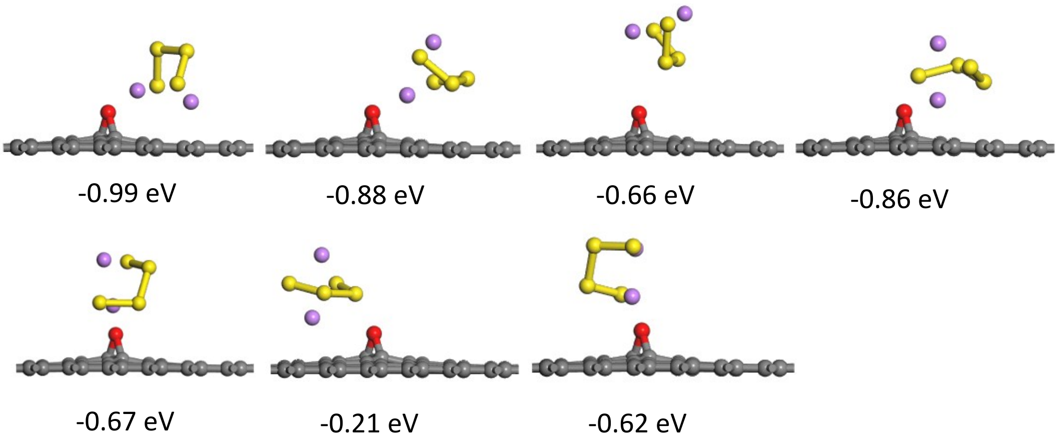
-0.99 eV
-0.88 eV
-0.66 eV
-0.86 eV
-0.67 eV
-0.21 eV
-0.62 eV

(f)

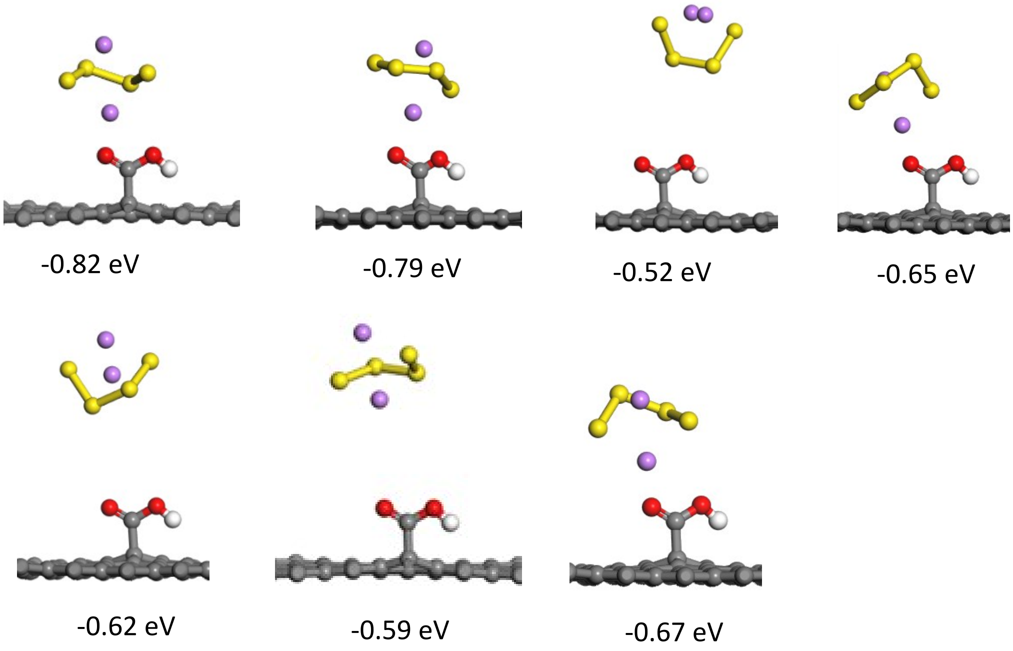

Fig. S1 Different configurations and corresponding adsorption energies of Li_2S_8 and Li_2S_4 on hG (a,d), eG (b,e) and cG (c,f)

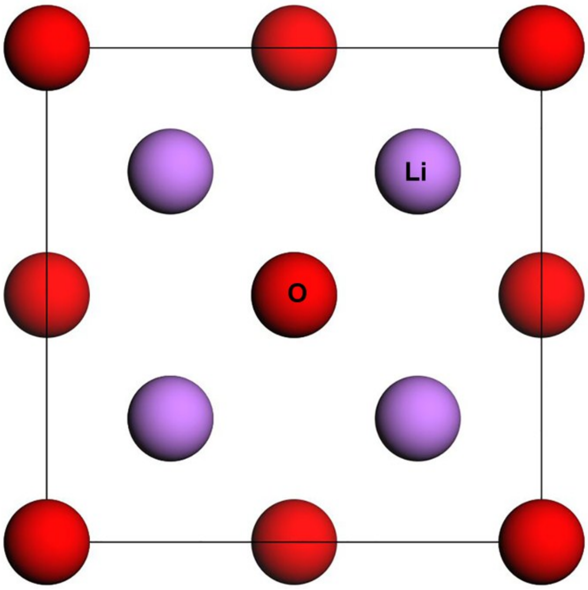

Fig. S2 Optimized structure of Li_2O

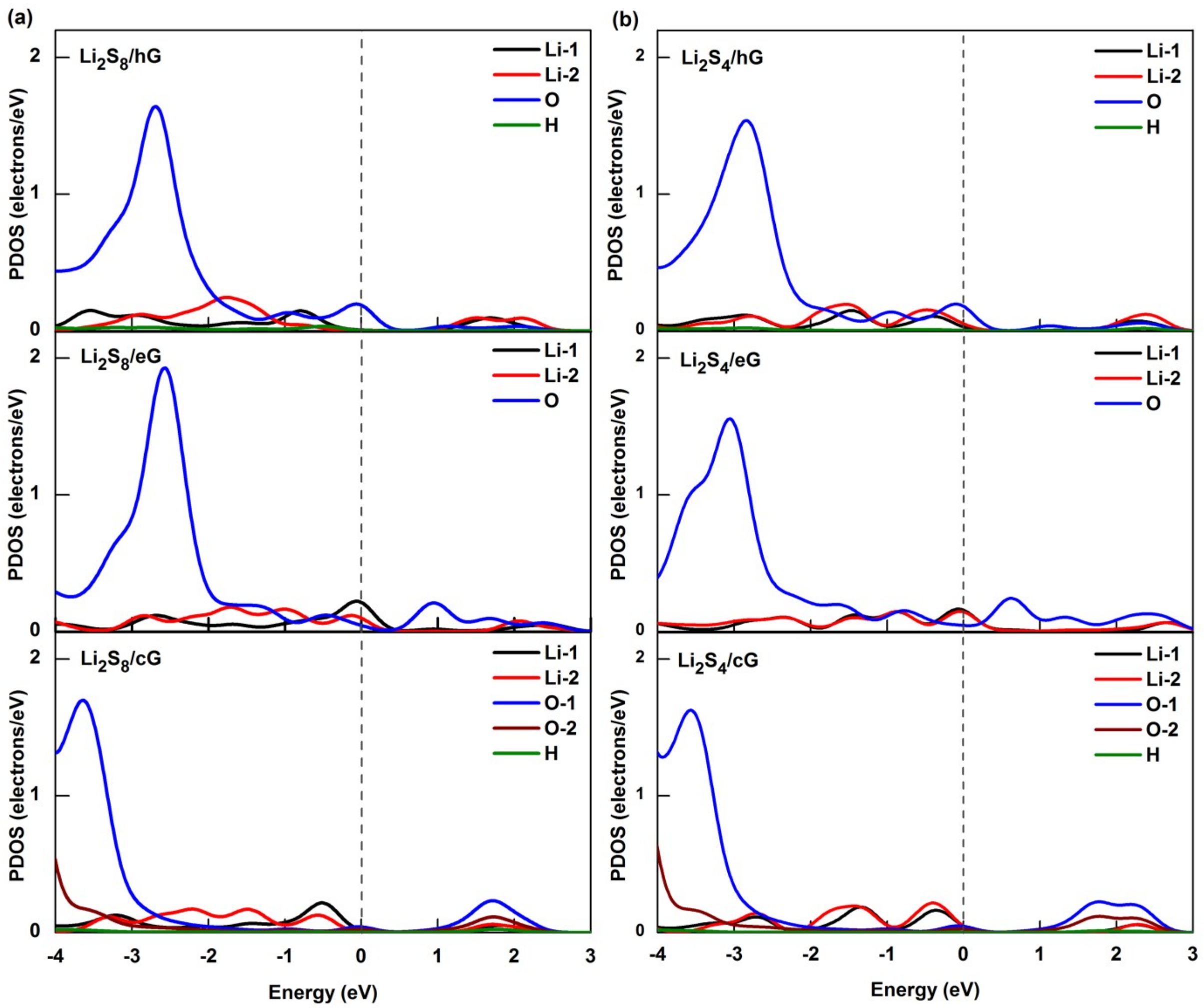

Fig. S3 Atomic partial density of states near the Fermi energy region for (a) Li2S8 and (b) Li2S4 on hG, eG and cG

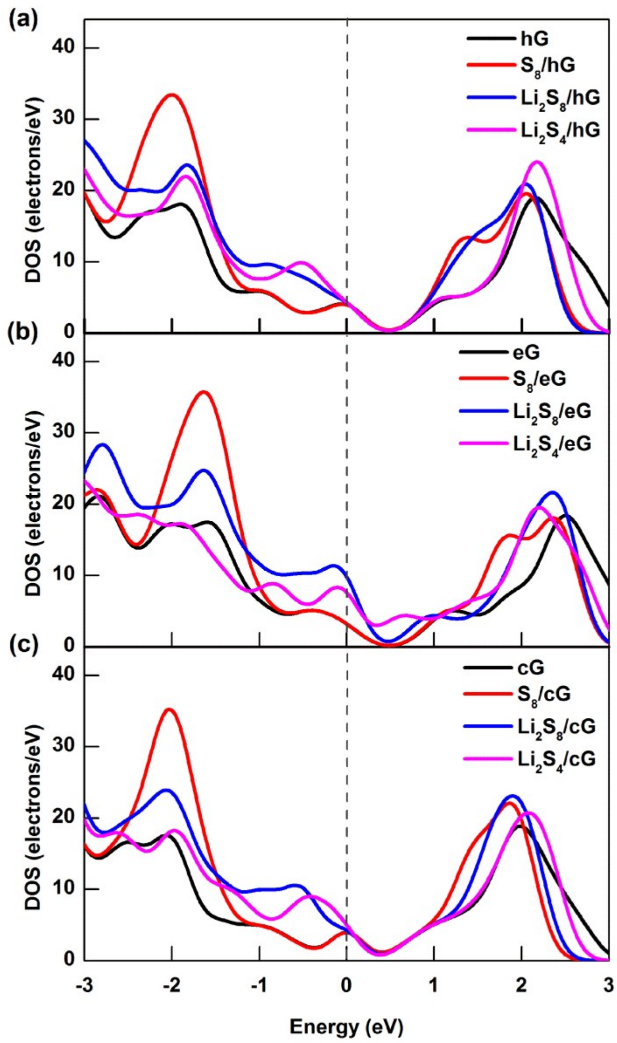

Fig. S4 Total density of states near the Fermi energy region for S8, Li2S8 and Li2S4 on a) hG, b) eG and c) cG

Table S1 Energy components related to adsorption energies of S_8, Li_2S_8 and Li_2S_4 to microporous graphene

Pore Size (Å)	$E_{graphene}$ (eV)	E_S where S = S_8 (eV)	E_{Total} (eV)	E_{ads} (eV)
7.5	-74621.756	-86654.006	-161276.987	-1.22
10	-74621.601	-86654.006	-161276.332	-0.71
12.5	-74621.595	-86654.006	-161276.244	-0.64
15	-74621.584	-86654.006	-161276.210	-0.62
17.5	-74621.525	-86654.006	-161276.126	-0.60
20	-74621.516	-86654.006	-161276.118	-0.60
Pore Size (Å)	$E_{graphene}$ (eV)	E_S where S = Li_2S_8 (eV)	E_{Total} (eV)	E_{ads} (eV)
7.5	-74621.756	-87066.246	-161689.556	-1.55
10	-74621.601	-87066.246	-161688.871	-1.02
12.5	-74621.595	-87066.246	-161688.825	-0.98
15	-74621.584	-87066.246	-161688.726	-0.90
17.5	-74621.525	-87066.246	-161688.667	-0.90
20	-74621.516	-87066.246	-161688.496	-0.73

Pore Size (Å)	$E_{graphene}$ (eV)	E_S where S = Li_2S_8 (eV)	E_{Total} (eV)	E_{ads} (eV)
7.5	-74621.756	-87066.246	-161689.556	-1.55
10	-74621.601	-87066.246	-161688.871	-1.02
12.5	-74621.595	-87066.246	-161688.825	-0.98
15	-74621.584	-87066.246	-161688.726	-0.90
17.5	-74621.525	-87066.246	-161688.667	-0.90
20	-74621.516	-87066.246	-161688.496	-0.73

Table S2 Summary of adsorption energies between different substrates and S species

Substrate	Adsorption Energy (eV)		
	S_8	Li_2S_8	Li_2S_4
Graphene	-0.64	-0.93	-0.74
hG	-0.63	-1.23	-1.43
eG	-0.63	-1.08	-1.07
cG	-0.65	-1.29	-0.95

Table S3 Total DOS value at E_f of Sulfur species in different substrates

Species	Total DOS value at E_f (electrons/eV)
S_8/Graphene	4.32665
Li_2S_8/Graphene	9.33993
Li_2S_4/Graphene	6.48636
hG	3.98315
S_8/hG	3.99742
Li_2S_8/hG	4.23578
Li_2S_4/ hG	4.30917
eG	3.15841
S_8/eG	3.14733
Li_2S_8/eG	9.82511
Li_2S_4/eG	7.54303
cG	3.91877
S_8/ cG	3.91533
Li_2S_8/cG	4.24859
Li_2S_4/cG	4.93481

Table S4 Bond lengths after adsorption

	Li2S8-hG	Li2S8-eG	Li2S8-cG	Li2S4-hG	Li2S4-eG	Li2S4-cG
Li-O distance (A)	1.869	1.958	1.988	1.993	2.010	1.979
Li-S distance	2.476	2.477	2.476	2.463	2.449	2.435
